# First spike latency sensitivity of spiking neuron models

**DOI:** 10.1186/1471-2202-14-S1-P354

**Published:** 2013-07-08

**Authors:** Laura Trotta, Alessio Franci, Rodolphe Sepulchre

**Affiliations:** 1 Department of Electrical Engineering and Computer Science, University of Liège, Liège, 4000, Belgium; 2 Centre de recherché Inria Lille-Nord Europe, Parc scientifique de la Haute Borne, Villeneuve d'Ascq, 59650, France

## 

First spike latency is the long-lasting period preceding the first spike of a neuron submitted to a super-threshold stimulus. It has been suggested that this latency could code for stimulus recognition in several sensory systems [1,2].

**Figure 1:**
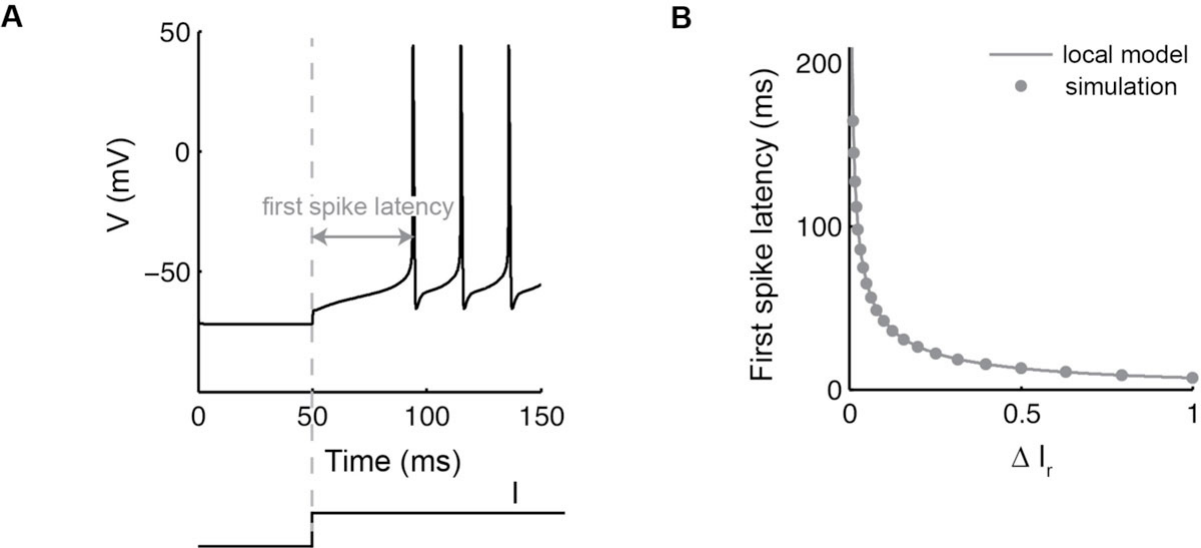
**First spike latency in **[4]**when the Type A-current conductance is set to a value which corresponds to regenerative excitability**. When the current is slightly above the saddle node, the neuron presents a robust first spike latency (A). First spike latency decreases with the relative variation of current ΔIr, i.e the distance to the saddle node bifurcation (B). Analytical predictions of a local model (solid line) fit the results obtained by numerical simulations of the full model (dots).

To encode information reliably, first spike latency should be sensitive to sensory inputs but robust to external perturbations. This paper studies the robustness of the first spike latency in spiking neuron models. These models are nonlinear and possibly high-dimensional. Understanding which parameters govern the first spike latency sensitivity in these systems generally requires extensive numerical simulations. We show that a local sensitivity analysis of the system is a good predictor of the global robustness of the latency code. This analysis, motivated by previous work on delayed decision-making in bistable models [3], relies on the assumption that the model posses a saddle node bifurcation with a sufficiently attractive center manifold. Results are illustrated on a 6-dimensional model including a K^+^-current of type A [4], a current of particular importance for first spike latencies, see Figure 1.

Our results show that first spike latency coding is particularly robust for a new type of excitability called regenerative excitability [5].
